# Comparison of out-of-pocket expenditure and catastrophic health expenditure for severe disease by the health security system: based on end-stage renal disease in South Korea

**DOI:** 10.1186/s12939-020-01311-3

**Published:** 2021-01-06

**Authors:** Sun Mi Shin, Hee Woo Lee

**Affiliations:** 1 Department of Nursing, Joongbu University, 201 Daehak-ro, Chubu-myeon, Geumsan-gun, Chungnam South Korea; 2 Hemodialysis Unit, Lee Hee Woo Internal Medicine Clinic, 1402, Gyebaek-ro, Seo-gu, Daejeon, South Korea

**Keywords:** National Health Insurance, Medical aid, Healthcare utilization, Out-of-pocket expenditure, Catastrophic health expenditure

## Abstract

**Background:**

Korea’s health security system named the National Health Insurance and Medical Aid has revolutionized the nation’s mandatory health insurance and continues to reduce excessive copayments. However, few studies have examined healthcare utilization and expenditure by the health security system for severe diseases. This study looked at reverse discrimination regarding end-stage renal disease by the National Health Insurance and Medical Aid.

**Methods:**

A total of 305 subjects were diagnosed with end-stage renal disease in the Korea Health Panel from 2008 to 2013. Chi-square, t-test, and ANCOVA were conducted to identify the healthcare utilization rate, out-of-pocket expenditure, and the prevalence of catastrophic expenditure. Mixed effect panel analysis was used to evaluate total out-of-pocket expenditure by the National Health Insurance and Medical Aid over a 6-year period.

**Results:**

There were no significant differences in the healthcare utilization rate for emergency room visits, admissions, or outpatient department visits between the National Health Insurance and Medical Aid because these healthcare services were essential for individuals with serious diseases, such as end-stage renal disease. Meanwhile, each out-of-pocket expenditure for an admission and the outpatient department by the National Health Insurance was 2.6 and 3.1 times higher than that of Medical Aid (*P* < 0.05). The total out-of-pocket expenditure, including that for emergency room visits, admission, outpatient department visits, and prescribed drugs, was 2.9 times higher for the National Health Insurance than Medical Aid (*P* < 0.001). Over a 6-year period, in terms of total of out-of-pocket expenditure, subjects with the National Health Insurance spent more than those with Medical Aid (*P* < 0.01). If the total household income decile was less than the median and subjects were covered by the National Health Insurance, the catastrophic health expenditure rate was 92.2%, but it was only 58.8% for Medical Aid (*P* < 0.001).

**Conclusion:**

Individuals with serious diseases, such as end-stage renal disease, can be faced with reverse discrimination depending on the type of insurance that is provided by the health security system. It is necessary to consider individuals who have National Health Insurance but are still poor.

## Background

In most countries, the economic gap between the rich and the poor has become more serious in modern society. If healthcare intervention is necessary to sustain the life of an individual, it is more likely that he/she will be faced with poverty. Therefore, strengthening health equity through inclusive and universal healthcare is an important health agenda in most countries. Health insurance has a positive effect on dispersing the risk of health financing for people, and the utilization of healthcare services has also increased. However, as social inequity increases, the level of catastrophic health expenditure (CHE) that each individual or household can afford is also decreasing [1–3]. The CHE is an indicator of the burden of healthcare expenditure and households’ capacity to pay [2]. The basic approach used to measure capacity to pay is based on consumption or income. Xu et al. [4] defined CHE as expenditure exceeding 40% of household consumption expenditure excluding subsistence expenditure (SE) [4]. On the other hand, Wagstaff & van Doorslaer [5] reported that prepaid income excluding that used for food consumption represents a household’s capacity to pay. Additionally, CHE is defined as out-of-pocket (OOP) medical expenditure that exceeds 2.5, 5, 10, 15 20, 25, 30% or 40% of a household’s capacity to pay [5].

Each country has a different type of health security system. The Korea National Health Insurance (NHI) system is a major health system that requires mandatory enrollment and involves the payment of premiums. On the other hand, the Korean Medical Aid (MA) system is operated by a national tax and local tax and acts as a national guarantee that all services used will be paid for. The NHI has been utilized by the entire population since 1989, and approximately 97% of Koreans, except those that use MA, are currently registered for this insurance [6–8]. However, people have to pay premiums every month to maintain the NHI. At this time, the monthly premium is determined by each individual’s level of income and property ownership. In other words, rich individuals have higher premiums than poor individuals, so the government has already introduced vertical equity. In addition, the government continues to maintain a policy to control medical expenses, thereby providing high-quality medical services at low prices. However, the NHI covers only approximately 56% of the total healthcare costs related to the utilization of healthcare services [9]. The remaining 44% must be paid at personal OOP expenditure, regardless of the individual's economic condition. Therefore, the logic of horizontal equity can be applied to personal copayment and excessive OOP expenditure for the poor can be considered as CHE.

Korea’s MA recipients, who account for only approximately 3% of the population, have the lowest incomes or cannot maintain a job, and thus, this system guarantees the health rights of all people. MA recipients do not pay a monthly premium and when they use healthcare services, there is little or no OOP expenditure. Therefore, the largest group of beneficiaries of vertical equity does not pay a premium and are faced with a minimal personal cost. The nation chose to implement the MA program based on strict screening criteria such as assets, income, and the income of family members. Korea’s MA system has greatly contributed to an improvement in the basic health security of vulnerable groups such as low-income families since 1977. However, by applying tax reduction, Korea has been providing customized health insurance benefits since 2015 through the NHI, and as of 2019, the number of MA recipients was 1,489,000, a decrease of 19% from 1,841,000 in 2008 [6, 7]. This policy is based on the assumption that there will be no adverse effects even if a MA recipient qualifies for the NHI.

The implementation of different types of health security systems and different coverage rates have limited the use of healthcare services or created a moral hazard due to the overuse of unnecessary healthcare services. High copayments are utilized to limit the abuse of medical services and are in line with horizontal equity because everyone must pay for the services they need. Previous studies showed that the level of unmet healthcare needs was higher for NHI recipients than MA recipients [8, 10–12]. Additionally, even when they have the same disease, the healthcare utilization rate for MA recipients was higher than that for NHI recipients, and the gap continues to widen [13–16]. This problem occurs because how much each individual has to pay for medical services is determined by the health security system. However, if healthcare services are vital to sustain life, the healthcare utilization of NHI recipients cannot be further reduced. Additionally, if subjects have a serious illness, regardless of whether they have the NHI or MA, healthcare providers provide the appropriate treatment following standardized care guidelines. Therefore, OOP expenditure for serious diseases will vary depending on the type of healthcare coverage, and in some types, induce poverty.

In this study, subjects with a serious disease for which healthcare intervention is essential, namely, end-stage renal disease (ESRD), were studied to understand the financial burden associated with each type of health security system. ESRD refers to stage five chronic kidney disease (CKD) and requires dialysis, kidney transplantation, and conservative care. In Korea, for ESRD on hemodialysis (HD), the health care expenditure was $1561 million in 2014 and increased by 32.2% in 2009. The number of patients undergoing dialysis and kidney transplantation (KT) in Korea is 1446 per million people. Among them, 69.8% underwent HD, 10.0% underwent peritoneal dialysis (PD), and 20.2% underwent KT [17]. However, the gross expenditure will be larger because personal copayment is not included in the calculation. Even though ESRD is a serious high-cost illness in most countries, healthcare expenditure for this disease have not been determined. There have been few studies on OOP expenditure for individuals with ESRD.

Therefore, it is necessary to understand the financial burden of illness based on the types of health security system provided by the health security system. Few studies have assessed whether the rate of healthcare utilization of poor people and those are public guaranteed health benefits by the nation is equitable, and the general population has been required to pay insurance expenses to the NHI Corporation. This study aimed to compare the healthcare utilization, OOP expenditure, and CHE for Patients with ESRD with either the NHI or MA over a 6-year period (2008–2013) Korea Health Panel (KHP) data was obtained both cross-sectionally and longitudinally. This study estimated the reverse discrimination of individuals with ESRD with different types of health security system. In addition, this study provided basic data that can be used for international comparisons of the OOP burden of Patients with ESRD.

## Methods

### Design

Using data from 2008 to 2013, this study performed two analyses, one of which was a pooled time series cross-section analysis (N*T) and one of which was a longitudinal analysis of time-series data.

### Data source

This study used KHP data from 2008 to 2013 that were collected by the Korea Institute for Health and Social Affairs (KIHASA) and NHI Corporation. KHP data are nationally representative and appropriate for decision making about healthcare policy. The KHP uses an extraction framework for 90% of the 2005 census to maintain the national scale for the survey. Subjects for the KHP were selected according to the probability-proportional stratified sampling method and surveyed annually for the same variables since 2008. The baseline sample was 7866 households and 24,616 household members. However, in 2012, only 5856 households and 17,417 household members remained due to panel attrition, such as death or rejection of the investigation. Accordingly, approximately 2500 households were added nationwide and included in the survey starting with the 8th survey in 2013.

The KHP survey obtains data on approximately 500 variables. The survey collects information on the demographic and sociological characteristics of the survey participants, such as the households and household members’ assets and income per year, number of family members, type of health insurance, registration and type of disability; health care characteristics, such as diagnosed diseases, emergency room visits, outpatient visits, and hospitalization and copays per case, medication costs, satisfaction with medical care, and complementary medical use and costs; additional aspects, such as health behaviors and quality of life; and insurance information, such as the type of insurance utilized. The KIHASA and NHI Corporation generated household income deciles using the primary data: the total household income per year divided by the square root of the number of household members [4], which are classified as the 1st (minimum) decile and the 10th (maximum) decile. A sampling weight was applied considering the attrition rate of participants in the panel data. For this study, the Institutional Review Board (IRB) formally approved the use of KHP data (KIHASA 2016–01).

### Data collection

The panel data were collected annually by trained interviewers visiting homes and interviewing subjects face-to-face. To reduce recall bias for information about medical records, the subjects recorded OOP expenditure in the medical household account book with the reason for healthcare utilization immediately after visiting a clinic or hospital. The collection of receipts, the importance of quick records, and how to fill out a medical household account book are continuously educated through guide materials and counseling every year. In addition, the visited interviewer reviewed receipts and confirmed to determine if the records of medical households were accurate. In some cases, to improve accuracy, the actual hospital clam data were checked in a cross. Since it is data over a long period of time, the actual cost was recorded without applying the consumer price index so that even the price fluctuation of the medical service could be known. The collected data are released to researchers approximately 3 years after confirming that they are completed and have been verified through data entry editing, imputation, building weight, variance estimation, and trial data conference.

### Using variables

To the greatest extent possible, this study used raw data for the variables, such as gender, survey year, type of health insurance (NHI or MA), type of disability, number of comorbidities, number of visits to a care center: emergency room (ER) visits, outpatient department (OPD) visits and inpatient visits, OOP expenditure, and total household income decile.

Comorbidity refers to any chronic disease diagnosed by a medical doctor over the past year, such as hypertension, diabetes, hyperlipidemia, arthritis, tuberculosis, ischemic heart disease, and cerebrovascular disease. The various chronic diseases reported by the subjects were verified by a trained interviewer, who entered into a standardized disease code.

To date, medical expenditures have analyzed the cost of insurance coverage billed by each hospital. OOP expenditure for each individual has been rarely studied because insurance claims data do not represent individual OOPs. It is very useful to know the gross amount of healthcare expenditure. Therefore, KHP has investigated annual OOP expenditure to decide health policy. OOP expenditures are noninsured benefits or copayments. The copayment is that each person must pay after excluding insured coverage for ER, admission, OPD visits, and prescription drug purchases. Therefore, the copayment of dialysis, transplantation, and conservation care of ESRD were included in OOP expenditure. OOP expenditures were analyzed separately by using them in the ER, admission and OPD visits. Additionally, to determine the OOP expenditure of the drug, drug costs were added to the OOP expenditure of ER, admission and OPD visits, then referred to as personal total OOP medical expenditure. The newly created variable for this study using raw data was total household income per year; SE and CHE; a type of healthcare service: dialysis, KT and conservative care.

Originally, KHP data generated total household income per year by adding gross earned income and gross asset income in households. Household gross earned income is the sum of all household members’ earned income: months worked. Total asset income was the sum of real estate and property income, financial income, social insurance, private insurance, government subsidies, private subsidies, and other income. In this study, total household income per year was adjusted for household size according to the OECD’s square root index method [18].

KHP data investigated monthly average living expenses, which excludes savings. For the SE, only the food cost can be applied to apply the extreme poverty line. However, when applying the wide poverty line, food consumption can be applied [5]. In other words, SE was not standardized in all countries. Korea’s Ministry of Health and Welfare announced that SE is the minimum cost necessary to maintain a healthy and cultural life. Therefore, in this study, SE was defined as the cost of living after excluding saving, and then the consumption equivalence scale was applied to adjust the size of the household [18]. There are several equivalent methods, but we used the OECD square root index. This is a method of calculating equalized personal income by dividing household income by the square root of the number of household members.

In this study, the household capacity to pay was created by subtracting the SE from the total household income, which was adjusted for household size. Then, if it exceeded 40% of the household capacity to pay, it was defined as CHE. In addition, if the household capacity to pay was zero or a negative value, the person was defined as the medical poor [5].

The type of healthcare service, such as dialysis, KT and conservative care, was a newly created variable for this study. In the KHP data, the type of healthcare service or fee for service of each disease was not investigated. Therefore, we classified the type of healthcare service using disability type. It was classified as dialysis in case of dialysis-disabled by law; KT in case of KT-disabled by law; conservative service in case of absence of disability by law. This is because dialysis patients and KT are enrolled as Grade 2 and Grade 5 kidney-disabled, respectively, under the Disabled Welfare Act in Korea.

### Study subjects

Of the 111,869 KHP subjects from 2008 to 2013, 305 (0.28%) were diagnosed with ESRD (N18-N19 according to Korea Classification of Diseases-6 code) by medical doctors. When looking at 305 subjects by year, there were 34 in 2008, 47 in 2009, 56 in 2010, 60 in 2011, 52 in 2012, and 56 in 2013.

### Statistical analysis

This study conducted cross-sectional analysis and panel analysis. In pooled time series cross-section analysis, chi-square and t-tests were performed to compare demographic characteristics, CHE and the medical poor ratios between NHI and MA. In addition, ANCOVA confirmed total OOP expenditures due to ER, admission and OPD visits, and prescription between NHI and MA. At this time, gender, age, type of healthcare service, and comorbidities were used as covariables. Every OOP expenditure (South Korean Won, KRW) converted to USD ($) based on the exchange rate on July 1, 2008 (1$ = 1050.89 KRW) [19].

In the panel analysis, the total OOP expenditure trend of subjects for 6 years from 2008 to 2013 was identified. We built three models: a saturated model with an unstructured covariance matrix, a saturated model with a compound symmetry covariance matrix, and the main effects model with a compound symmetry covariance matrix. Then, the final results were presented by the main effects model, which had the lowest Akaike’s information criterion and Bayesian information criterion.

The statistical test was performed after excluding missing data for each variable using SAS 9.4 (SAS Institute Inc., Cary, NC, USA). *P* values of less than 0.05 were regarded as statistically significant.

### Definitions of terms

#### NHI and MA among health security system

In Korea, the health security system has NHI and MA. The NHI is an obligatory system for the national people. The people have to pay an insurance fee to NHI Cooperation and then can receive medical benefits if they need it. Their copayment of individuals has been from 20 to 60% of the total medical fee for each medical service [10, 11]. They pay copayment in admission approximately 20% of the total fee, and copayment in OPD visits approximately 30% ~ 60% of the total fee [11]. In this study, 237 subjects had NHI.

Meanwhile, MA is a public healthcare assistance program that supports the lowest income group or the person with an incapacity for maintaining their life. Their medical expenses are covered by the national tax and local tax under government responsibility. MA consists of type 1 and type 2. MA type 1 includes persons who are poor and the incapacity of working. MA subjects do not pay any copayment in admission and do pay copayment ($0.95 or $1.93) at only OPD visits [11]. MA type 2 includes people who are poor but can work, different from MA type 1. There were 68 subjects with MA in this study, which consisted of type 1 (*n* = 55) and type 2 (*n* = 3), but we did not classify them separately because of the small number of subjects (Table 1, Table 2).

**Table 1:** Representative programs of the Korean Health Security System

Criteria	NHI^**a)**^	MA^**b)**^
System	Social insurance	Public assistance (type 1, type 2)
Subject	All people except those with MA (Mandatory subscription)	Selected people having difficult living
Finance method	Premiums and treasury	Tax
Operation & management	Nation & public corporation	National & local governments
Insurance premium burden	Based on income level	No premium
Insurance benefits	Uniform	Vary
Population (2020)	97.2%	2.8%
Copayment		
Admission	20% of total medical expenses	None
OPD	60% ~ 30% of total medical expenses	Approximately $ 0.95 ~ $ 1.93 type 1: 5% of special equipment service fee type 2: 15% of special equipment service fee & 15% of medical benefit costs when using secondary or tertiary medical institutions

^a)^National Health Insurance

^b)^Medical Aid

## Results

### Sociodemographic characteristics of the subjects

Among a total of 305 subjects for 6 years, 237 subjects (77.7%) had NHI, and 68 subjects (22.3%) had MA. By year, 34 subjects were in 2008, 47 in 2009, 56 in 2010, 60 in 2011, 52 in 2012, and 56 in 2013. Males were only 41.8% in NHI but 63.2% in MA. The mean age of subjects with NHI was 61.8 years and MA was 53.9 years. The subjects at and over 65 years were 46.4% in NHI and only 19.1% in MA. In terms of healthcare service type, dialysis (*n* = 128) was 39.2 and 51.5% in NHI and MA, renal transplantation (*n* = 21) was 8.0 and 2.9% in NHI and MA, and conservative treatment (*n* = 156) was 52.7 and 45.6% in NHI and MA, respectively. There was no significant difference. The average decile of total household income (lowest 0 to highest 10) was 4.5 in NHI and 2.6 in MA (*p* < .0001). There were no significant differences in the number of chronic diseases, with 4.5 in NHI and 5.0 in MA (Table 2).

**Table 2:** Sociodemographic characteristics of the study subjects for a 6-year period

Classification	Total		NHI^a)^		MA^b)^		t or χ^2^ score	*p*-value
	n	%	n	%	n	%		
All	305	100	237	100	68	100		
Age ± SD	60.1 ± 13.6		61.8 ± 13.5		53.9 ± 12.2		4.63	< 0.0001
Gender								
Male	142	46.6	99	41.8	43	63.2	9.78	0.002
Female	163	53.4	138	58.2	25	36.8		
Age								
< 65 years	182	59.7	127	53.6	55	80.9	16.36	<.0001
≥65 years	123	40.3	110	46.4	13	19.1		
Survey year								
2008	34	11.2	26	11.0	8	11.8	0.98	0.964
2009	47	15.4	37	15.6	10	14.7		
2010	56	18.4	43	18.1	13	19.1		
2011	60	19.7	46	19.4	14	20.6		
2012	52	17.1	39	16.5	13	19.1		
2013	56	18.4	46	19.4	10	14.7		
Type of healthcare service^c)^								
Dialysis (HD or PD)	128	42.0	93	39.2	35	51.5	4.39	0.11
Kidney transplantation	21	6.9	19	8.0	2	2.9		
Conservative care	156	51.2	125	52.7	31	45.6		
Total household income decile ±SD	4.1 ± 2.7		4.5 ± 2.8		2.6 ± 1.6		7.36	<.0001
Number of comorbidities ±SD	4.6 ± 2.7		4.5 ± 2.6		5.0 ± 3.0		−1.24	0.21

^a)^National Health Insurance

^b)^Medical Aid, type 1 for individuals incapable of working (*n* = 55) and type 2 for those capable of working (*n* = 13)

^c)^In terms of the type of healthcare service, dialysis indicates that the patient had already been registered for dialysis by law, and kidney transplantation indicates that the patient had already been registered for kidney transplantation by law. However, conservative treatment indicates that the patient had ESRD but was not subject to registration by law

### Healthcare utilization rates for the NHI and MA programs

The proportion of annual healthcare utilization for pooled 6-year data was identified in NHI and MA; 24.1 and 27.9% in the ER visits; 39.2 and 50.0% in admission; 99.2 and 98.5% in OPD visits, respectively. There were no significant differences. In addition, the annual mean frequency of healthcare utilization in NHI and MA was 1.7 and 1.6 times in ER visits, 2.0 and 2.1 times in admission, and 75.2 and 92.2 times in OPD visits, respectively. There were also no significant differences (Table 3).

**Table 3:** Annual healthcare utilization for the NHI and MA programs for the 6-year study period

Classification	NHI^a)^ *n* = 237 (%)	MA^b)^ *n* = 68 (%)	χ^2^ score	*p*-value
			F score	
Emergency-room (*n* = 76)				
Visit rate (%)	57 (24.1)	19 (27.9)	0.43	0.513^*)^
Number of visits ±SE^c)^	1.7 ± 0.2	1.6 ± 0.4	0.08	0.701^*)^
Admission (*n* = 127)				
Admission rate (%)	93 (39.2)	34 (50.0)	2.52	0.113^*)^
Number of admission ±SE^c)^	2.0 ± 0.2	2.1 ± 0.3	0.55	0.816^*)^
Outpatient department visits (*n* = 302)				
Visit rate (%)	235 (99.2)	67 (98.5)	0.21	0.644^*)^
Number of visits ±SE^c)^	75.2 ± 62.6	92.2 ± 71.9	0.25	0.620^*)^

^a)^National Health Insurance

^b)^Medical Aid, Medical Aid type 1 (*n* = 55) and Medical Aid type 2 (*n* = 13)

^c)^Adjusted for gender, age, type of healthcare service, and number of comorbidities

^*)^The p-value for ho was > = 0.05 (ho: The utilization rate or frequency is the same for the NHI and MA programs)

### Means of annual OOP expenditure according to NHI and MA

Using data for 6 years from 2008 to 2013, we compared the annual means of OOP expenditure in ER, admission and OPD visits between NHI and MA. The results were similar to those at admission. $2020.8 and $692.1 (2.9 times higher in NHI than MA) (*P* = 0.01), and in OPD visits, $1120.6 and $290.2 (3.9 times higher in NHI than MA) (*P* < 0.001). Additionally, the total annual OOP expenditure was $2154.2 and $657.5 in NHI and MA, respectively (3.2 times higher in NHI than MA) (*P* < 0.001). Thus, the expenditure of NHI was several times higher than that of MA (Table 4).

**Table 4:** Means of annual out-of-pocket expenditure ($^a)^) by ANCOVA^b)^ for pooled 6-year data

Classification	NHI^c)^	MA^d)^	F score	*p*-value	NHI:MA
	Mean ± SE	Mean ± SE			
OOP medical expenditure					
Emergency room visits (*n* = 76)	141.7 ± 34.1	34.2 ± 78.7	1.34	0.250	4.1:1
Admission (*n* = 127)	2020.8 ± 251.8	692.1 ± 432.0	6.11	0.01	2.9:1
Outpatient department visits (*n* = 302)	1120.6 ± 75.1	290.2 ± 145.5	24.7	< 0.001	3.9:1
Total out-of-pocket expenditure (*n* = 304) ^e)^	2154.2 ± 142.2	657.5 ± 274.5	22.4	< 0.001	3.2:1

^a)^South Korean Won (KRW) converted to USD ($) based on the exchange rate on July 1, 2008

^b)^Adjusted for gender, age, type of healthcare service, and number of comorbidities

^c)^National Health Insurance

^d)^Medical Aid, Medical Aid type 1 (*n* = 55) and Medical Aid type 2 (*n* = 13)

^e)^Includes expenditure for emergency room visits, admissions, outpatient department visits and prescribed drugs

### Six-year trend of total OOP expenditure by panel analysis

**Fig. 1:**
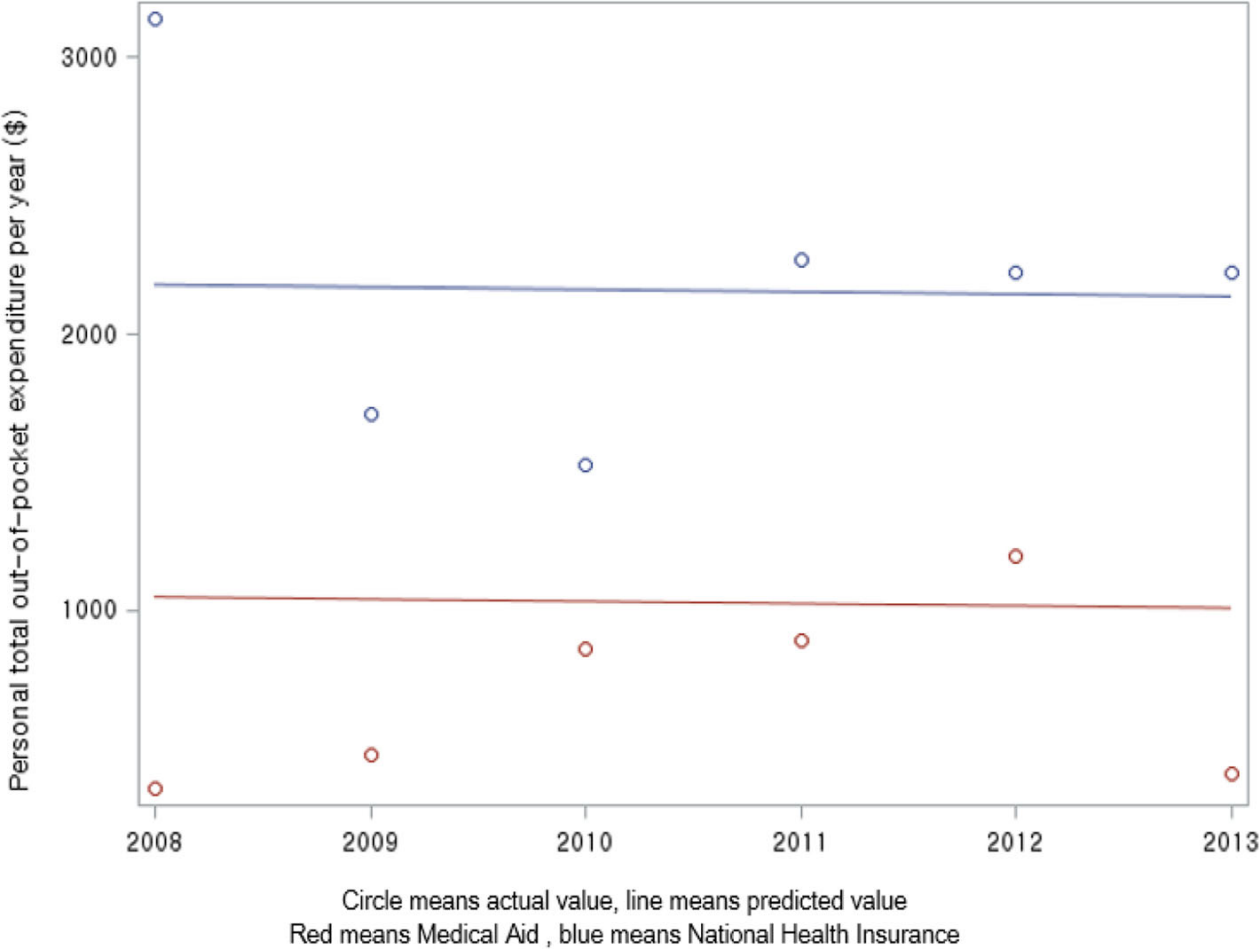
Personal total out-of-pocket expenditure by health security system^1). 1)^ Includes expenditure for emergency room visits, admissions, OPD visits, and prescribed drugs. There was a difference in the personal total out-of-pocket expenditure for individuals with the NHI and those with MA over the 6-year period (*P* < 0.01)

This study analyzed a 6-year period from 2008 to 2013 for total OOP expenditure according to NHI and MA. The subjects with NHI had more expenditure over a 6-year period than those with MA (*P* < 0.001). However, there was no significant difference in NHI or MA over a 6-year period (*P* = 0.926) (Table 5, Fig. 1).

**Table 5:** Trend for personal total out-of-pocket expenditure^a, b, c)^ for individuals who qualified for either the NHI or MA

Classification		Year						F/*p*-value of 6-year period	
		2008	2009	2010	2011	2012	2013	Between NHI and MA	Within NHI or MA
NHI^d)^ (*n* = 236)	Subjects	26	36	43	46	39	46	8.13/0.005	0.01/0.911
	Mean ± SE	2963.6 ±683.9	1738.9 ±333.1	1537.1 ±222.7	2339.1 ±350.4	2428.5 ±295.2	2228.1 ±331.7		
MA^e)^ (*n* = 68)	Subjects	8	10	13	14	13	10		
	Mean ± SE	927.0 ±1365.9	375.2 ±683.6	838.7 ±416.5	663.1 ±655.2	569.5 ±532.7	378.8 ±779.9		

^a)^Including expenditure for emergency room visits, admissions, outpatient department visits, and prescribed drugs

^b)^South Korean Won (KRW) converted to USD ($) based on the exchange rate on July 1, 2008

^c)^Adjusted for gender, age, type of healthcare service, and number of comorbidities

^d)^National Health Insurance

^e)^Medical Aid, Medical Aid type 1 (*n* = 55) and Medical Aid type 2 (*n* = 13)

### Comparison of catastrophic health expenditure for the NHI and MA programs

To determine the relationship between OOP expenditure and CHE, the household capacity to pay was calculated after adjusting for the total household income and SE according to the OECD equivalence scale. In group 1, there were no significant differences in CHE for 62.1 and 58.8% in NHI and MA, respectively. However, CHE was 92.2 and 58.8% among the NHI under the median of the total household income decile and MA, respectively. The medical poor, in which the household’s capacity to pay became more negative, was 21.5% in NHI and 16.2% in MA. In addition, among households with under median household income decile, the medical poor was 34.4% (Table 6).

**Table 6:** Comparison of the CHE prevalence for individuals with the NIH and those with MA

Criteria	Group 1 (*n* = 305)					Group 2 (*n* = 136)				
	All NHI		All MA		F or *X*^2^ /*p*-value	NHI for less than the median of total household income decile		All MA		F or *X*^2^ /*p*-value
	n	Mean ± SE or %	N	Mean ± SE or %		n	Mean ± SE or %	n	Mean ± SE or %	
Total household income per year^a), b)^	232	15,905.6 ± 560.9	68	7356.4 ± 1072.6	47.8 /<.0001	64	7037.8 ± 503.8.	68	8354.1 ± 485.4	2.88 /0.09
Subsistence expenditure^a), c)^	237	11,951.6 ± 356.4	68	6809.1 ± 689.7	42.0 /<.0001	68	7034.3 ± 362.6	68	7210.8 ± 6493.4	0.10 /0.76
Household capacity to pay^a), d)^	232	3803.7 ± 368.6	68	478.3 ± 704.9.	16.8 /<.0001	64	215.9 ± 278.1	68	1173.5 ± 268.0	10.53 /0.002
Annual total OOP expenditure^a), e)^	237	2145.8 ± 141.8	68	655.1 ± 274.3	22.32 /<.0001	68	1920.7 ± 1549.7	68	617.3 ± 246.3	19.62 /<.0001
Prevalence of CHE^f)^	144	62.1	40	58.8	0.23 /0.63	59	92.2	40	58.8	19.57 /<.0001
Medical Poor^g)^	50	21.5	11	16.2	0.94 /0.62	22	34.4	11	16.2	20.70 /<.0001

^a)^South Korean Won (KRW) converted to USD ($) based on the exchange rate on July 1, 2008; adjusted for gender, age, type of healthcare service, and number of comorbidities

^b)^Added gross earned income and gross asset income for households and adjusted for household size using a consumption equivalence scale

^c)^Minimum cost of maintaining a healthy and cultural-appropriate life, excluding savings

^d)^Household capacity to pay refers to the income that a household can actually use after excluding subsistence expenditure

^e)^Included expenditure for emergency room visits, admissions, outpatient department visits and prescribed drugs

^f)^OOP expenditure exceeds 40% of the household capacity to pay

^g)^The household capacity to pay is zero or a negative value; the negative value becomes larger when the copayment is paid

## Discussion

Regardless of the difference between the rich and the poor, the right to human health must be guaranteed. To this end, most countries are implementing various policies to ensure the health of the people. In particular, in Korea, there is an NHI system that practices universal medical care through horizontal equity that emphasizes the health rights of all people. This is a system that prevents people from falling into poverty due to medical care, as medical services are guaranteed through insurance premiums paid by the people. On the other hand, it has an MA system for the inclusive health of low-income families through vertical equity. MA is a public assistance program that supports the right to health of people without living ability under the responsibility of the nation and local governments. The selection of MA beneficiaries is for persons recognized by the Minister of Health and Welfare as needing medical benefits, recipients for basic life security, people without family guards, homeless people, disaster victims, adoptive children, etc. MA is a social safety net that resolves poverty after health problems happen without paying premiums.

Actually, resources are finite. Health policy for vertical equity requires financial resources and may be unfair from the standpoint of certain classes that value horizontal equity. In other words, new blind spots and reverse discrimination can emerge with the reinforcement of inclusive medical care that emphasizes vertical equity. However, scientific evidence for these health care issues is important for policy development. Health equity, fairness, and the relationship between poverty and health must be explored in a variety of ways. One of them is the difference in OOP expenditure under the health care system. In particular, ESRD is a high-cost chronic disease that requires kidney replacement therapy, such as HD, PD, and KT. Therefore, it prioritizes the life-sustaining effect as a disease serious enough that the efficiency cannot be considered. However, few studies have been performed on OOP expenditure on ESRD, especially the comparison of NHI and MA. In addition, most Patients with ESRD need lifelong treatment. Therefore, it is important to understand the OOP tendency of ESRD by the health security system.

In this study, 305 (0.27%) out of 111,869 subjects in the 6-year panel data were diagnosed with ESRD. The prevalence per 1 million was 2726, which was higher than the prevalence of 1446 Koreans published in 2013. This can be inferred because of the basic nature of the panel data that oversamples the vulnerable person such as low-income and disabled individuals. Among them, 77.7% belong to NHI and 22.3% belong to MA, which was a much higher distribution than only approximately 3% of MA among the Korean population. It could be interpreted that ESRD subjects are frail enough to warrant protection by the country. The elderly subjects accounted for 46.4% of the NHI, and the MA accounted for only 19.1%. The mean age of subjects with MA was 53.9 years, which was younger than 61.8 years of NHI. The reason why MA subjects are relatively younger can be thought of as a large number of innately vulnerable people, including genetic disorders, disabled, homeless and adopted persons. Therefore, people in MA can live longer in ESRD status than in Individuals who utilized the NHI. Additionally, the common causes of ESRD are chronic diseases such as diabetes and high blood pressure. It will increase due to aging in most societies [17, 20]. Therefore, we should also consider preventive strategies to ease the burden on individuals and countries due to ESRD.

For a severe disease such as ESRD, healthcare utilization did not differ significantly between NHI and MA. This was a different result from the previous study. In previous studies, including all diseases, MA subjects were using more health care than NHI beneficiaries. This was because the burden of copayment was relatively lower than that of NHI. They used 1.4 times more OPD and 1.6 times more admission [13–15]. In particular, patients with NHI but low income had significantly lower medical use than patients with MA [8, 21–24]. In contrast, in this study, there was no significant difference in the rate and frequency of healthcare utilization in ER, admission and OPD visits between NHI and MA. Therefore, in the case of serious diseases such as ESRD, health care can no longer be reduced regardless of the type of healthcare system. The treatments they receive are essential for survival.

Regardless of the equivalent healthcare utilization, annual OOP expenditure was significantly unequal between NHI and MA. Subjects with NHI had more OOP expenditure than MA. The OOP expenditure of admission was 2.9 times ($2020.8 vs. $692.1) (*P* = 0.01) and that of OPD visits was 3.9 times ($1120.6 vs. $290.2) (*P* < 0.001). Additionally, ESRD subjects with NHI expended more OOP expenditure over 6 years than those with MA (*P* = 0.005). In addition, in a previous study that included all diseases, the OOP expenditure of NHI was 1.5 times that of MA, which was relatively lower than that of ESRD disease [8]. Thus, subjects with a serious disease such as ESRD with NHI faced a much greater economic burden than those with MA. Furthermore, subjects with high OOP expenditure are more likely to face the risk of CHE [25–28]. CHE was compared between subjects with NHI under the median of household income decile and with MA. CHE was 92.2 and 58.8% among the NHI and MA, respectively. Additionally, the medical poor, in which the household’s capacity to pay became more negative, was 34.4% in NHI and 16.2% in MA.

The purpose of this study was to identify the actual burden on ESRD because the cost studies in the previous have been based on insurance claims data [17, 29–34]. The detailed meaning is as follows. First, in serious diseases such as ESRD, the NHI subject’s medical use was equivalent to MA. Second, in subjects with NHI, OOP expenditure was higher and more CHE than MA. Third, the higher OOP expenditure burden in NHI than MA was identified over a 6-year trend from 2008 to 2013. Higher OOP expenditure has been reported to have disadvantages that impede healthcare utilization when inevitably seeking health care [35–39]. Therefore, Individuals who utilized the NHI with a severe disease, such as ESRD, have a greater financial burden than MA subjects. Heath policies should be able to provide more medical care to those who need more medical care [40]. We noted the possible factors leading to this discrimination. Korea has recently emphasized universal and inclusive medical welfare. However, unlike this phenomenon, the number of recipients of MA has gradually decreased in Korea. In 2006, it was 1,711,076 people, which was 3.8% of Korea’s total population. In 2020, it is 1,496,000, which is 2.8% of the nation. Approximately 215,000 people fewer people use MA [11, 14] because there has been a transformation in the MA system to stabilize national healthcare finances. Therefore, people who have to be covered by MA are likely to have the NHI and face an increase in CHE.

Healthcare policymakers should consider mitigating the medical burdens of those who belong to the NHI but are poor. Suggestions for this study are as follows. First, in the selection criteria of MA, not only the subject’s property and workability are considered but disease requiring long-term treatment and CHE should be used as selection indicators. Second, if the size of MA cannot be increased according to the policy, if it belongs to NHI but is medical poor, it is necessary to find a way to pay health subsidies. Third, for those who have not yet converted from CKD to ESRD, it is necessary to seek a program to prevent further progress by being registered by an academic society or public interest social group. In addition, a new prevention policy model, such as the payment of prevention incentives to the subject, could be considered.

Although this study aimed to identify OOP expenditure and CHE of serious diseases such as ESRD according to the health security system, there are many limitations. First, in this study, OOP expenditure was not analyzed as a fee for service of ESRD but integrated expenditure on ESRD. This is because the fee for services such as dialysis, KT and conservative care of ESRD could not be distinguished from the raw data. Second, the data of this study have accumulated data of relatively rare ESRD over the years, and despite a large number of survey variables, the patient’s disease history could not be explored. It is necessary to explore inequity according to disease progression. Third, this study found the reverse discrimination of ESRD by the health security system, but it was the result of not controlling household income at baseline. Therefore, the causal relationship between disease and poverty has not been revealed. If the number of subjects who are included is greater and the follow-up period is longer, an attempt to determine causal relationships is necessary. Fourth, the OOP expenditure of this study excluded indirect costs such as transportation and salary stoppages. Therefore, it must be estimated to be less than the actual cost of health care. Fifth, although the subject of ESRD is characterized by a relatively rare and serious disease, it is not possible to exclude the possibility of type 2 error because of the small size of the subject.

## Conclusion

Strengthening health equity is an important health agenda in most countries. In Korea, the healthcare utilization of patients with ESRD that have the NHI or MA was the same because of the severity of the disease. Meanwhile, data for a 6-year period showed that OOP expenditure for the NHI program was consistently higher than that of the MA program. In other words, healthcare services provided to individuals with severe diseases such as ESRD who utilized the NHI could not be reduced even though their costs were higher than individuals who utilized MA. As a result, Individuals who utilized the NHI have larger CHEs than MA subjects. This study is significant because it identified inequity among individuals with the NHI and those with MA who had a serious disease such as ESRD. Therefore, healthcare policymakers should consider reducing the medical burdens of individuals who utilize the NHI and are poor.

## Data Availability

The datasets used during the current study are available from the corresponding author on reasonable request. The data information found to the following link sites: https://www.khp.re.kr:444/eng/main.do

## Abbreviations

CHE: Catastrophic health expenditure
SE: Subsistence expenditure
OOP: Out-of-pocket
NHI: National Health Insurance
MA: Medical Aid
ESRD: End-stage renal disease
CKD: Chronic kidney disease
KT: Kidney transplantation
HD: Hemodialysis
PD: Peritoneal dialysis
KHP: Korea Health Panel
KIHASA: Korea Institute for Health and Social Affairs
ER: Emergency room
OPD: Outpatient department
KRW: South Korean Won
